# Perceptual distortions and deceptions: what computers can teach us

**DOI:** 10.1192/pb.bp.115.052142

**Published:** 2017-02

**Authors:** Matthew M. Nour, Joseph M. Nour

**Affiliations:** 1 Imperial College London; 2 King's College London; 3 Oxford University Clinical Academic Graduate School (OUCAGS), Oxford

## Abstract

The nature of perception has fascinated philosophers for centuries, and has more recently been the focus of research in psychology and neuroscience. Many psychiatric disorders are characterised by perceptual abnormalities, ranging from sensory distortions to illusions and hallucinations. The distinction between normal and abnormal perception is, however, hard to articulate. In this article we argue that the distinction between normal perception and abnormal perception is best seen as a quantitative one, resting on the degree to which the observer's prior expectations influence perceptual inference. We illustrate this point with an example taken from researchers at Google working on computer vision.

No mental state examination would be complete without a statement relating to abnormalities of perception. According to Fish, perceptual abnormalities may be classified as either sensory distortions (e.g. hyperacusis and dysmegalopsia) or sensory deceptions (e.g. illusions and hallucinations), with both categories deviating from veridical perception.^1^ The clean simplicity of this definition underlies part of its clinical utility, but also gives the impression that the characteristics of ‘normal’ veridical perception are well understood. The nature of perception, however, has troubled philosophers for centuries,^2,3^ and has been the focus of intense investigation by neuroscientists and psychologists in recent decades.^4–7^ Just how does the brain transform the light hitting the retina into the infinitely complex three-dimensional world that we see when we open our eyes? How much of what we perceive is really present in the sensory data hitting our eyes, and to what extent do our prior expectations shape our perception? Do we perceive the world as it really is, and if not, does that mean that our normal perceptions are distorted or deceptive? If so, in what way does normal perception differ from abnormal perception? These questions may seem to be only of philosophical relevance, but researchers working in the fields of perceptual neuroscience and computer vision are regularly confronted by them.

In this article we outline some exciting insights into how the brain may construct reality. Intriguingly, these findings have come from the field of machine learning, a branch of computer science and robotics.

## The problem of perception

How does your visual system construct a representation of the world? Perhaps most readers would reply that the brain extracts the information about the physical world that is contained within incoming sensory signals. Much of the neuroscience of the past half-century has investigated perceptual processing starting from this assumption.^4,8^ The account of perceptual processing found in most undergraduate textbooks states that the sensory processing pathway (including the sensory epithelia, subcortical nuclei, thalamus, sensory cortices and heteromodal association cortices) extracts information from incoming sensory data in a stepwise manner. If all goes smoothly we perceive the world ‘as it really is’.

**Fig 1:**
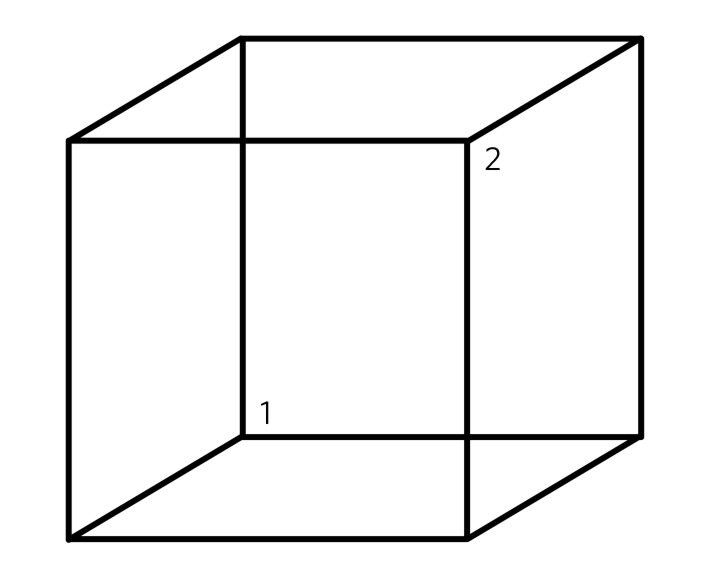
The Necker Cube illusion is a bistable visual illusion. The same sensory data are able to support two perceptual inferences (one in which corner 1 is closest to the observer, the other in which corner 2 is closest).

There are several problems, however, with the view that perception proceeds by extracting information from the incoming signals alone. Perhaps the most damning consideration was recognised by George Berkeley in the 18th century as the ‘inverse optics’ problem,^2,4^ which states that information collected by the sensory epithelia is insufficient to allow an unambiguous mapping back on to real-world sources. The light hitting the retina, for example, forms a two-dimensional image, which has an infinite number of possible three-dimensional ‘real-world’ sources. The image conflates information about object illumination, reflectance and transmittance.^4^ Computer vision faces similar problems. A car, for example, looks different from different viewing angles and distances, and in different lighting conditions. The problem of inferring the state of the world from sensory data alone is (mathematically) ill-posed.^9^ One powerful illustration of this principle is demonstrated by the famous Necker cube illusion,^5^ where the sensory information alone is insufficient to resolve the question of the orientation of the wire cube and there is no simple mapping between sensory data and perception (Fig. 1).

## Expectation is necessary for perception

If sensory data alone cannot support perception, how does the brain create accurate perceptual representations of the world? It is now appreciated that ill-posed problems such as vision can be made tractable by using contextual information to impose constraints on the interpretation of ambiguous data. In the case of vision, for example, past experiences of seeing similar visual scenes sets up expectations about the likely current state of the world, and any new sensory data are interpreted in light of these expectations. Consistent with this hypothesis, the sensory processing pathways in the brain do not just support one-way ‘bottom-up’ information flow (from low-level data in the primary sensory cortex to more complex representations in heteromodal association cortices), but also support ‘top-down’ information flow, whereby information about the current expected state of the world cascades down from high cortical areas to influence information processing in low sensory areas.^9,10^ Some have argued that the ‘heavy lifting’ of perceptual processing is performed by these top-down pathways, which make predictions about the state of the world that are tested against sensory data.^10^

The ability of the brain to make sense of sensory information has inspired computer scientists working on computer vision and similar problems to take a similar approach. Recently, researchers at Google created an impressive visual recognition system using a processing architecture inspired by the human brain, called an ‘artificial neural network’ (ANN).^11^ ANNs consist of artificial ‘neurons’ that are organised into layers, reminiscent of the brain's hierarchical organisation. These networks are particularly good at detecting features and patterns in new data, and using these features to perform classification tasks. This is similar to what the brain does when engaged in perceptual inference, which may be thought of as the detection of objects and meaningful patterns in sensory data. New data enter the ANN at the lowest ‘input’ layer (analogous to light hitting light-sensitive neurons in the retina) and is processed sequentially by progressively higher layers of the network. At each layer the network attempts to extract patterns and features from these data, with higher layers of the network extracting increasingly more abstract features. At the highest layer a ‘decision’ is made about what the data represent.

Importantly, a new ANN must be adequately ‘trained’ before it can perform successful pattern detection and classification tasks. During training the network is exposed to many different data-sets (e.g. images) and attempts to classify the data appropriately. The network is able to self-calibrate, guided by its successes and errors, in a process analogous to synaptic plasticity. After exposure to thousands of images of, say, cats, the network learns to recognise cats in images to which it has never before been exposed.

The well-trained ANN is primed to recognise salient features and patterns in new data in much the same way that the brain is primed to recognise the patterns in sensory data that are most important for detecting behaviourally relevant objects. Prior to training, the ANN is essentially blind to meaningful patterns in new data. In both the well-trained ANN and the mature human visual system the final decision about what a new image represents is the product of a delicate balance between the information contained within the image itself and the readiness of the network to detect certain features within new data.^9,10^

## Tipping the balance

Perception is therefore the product of two sources of information: the sensory data and prior expectations about the sort of information that the sensory data contain. What happens, however, when prior expectations are given too much weight?

The Google researchers provide an intuitive example of the problems that inappropriately strong prior expectations can cause in their ANN.^11^ As mentioned previously, the highest layers of the ANN contain latent representations of objects that the network has been trained to see. The Google researchers asked a network trained to see bananas to detect and enhance ‘banana-like’ features in an image that contained only meaningless noise. This manipulation inappropriately weighted prior expectation relative to sensory data. The result was that the network was able to ‘perceive’ objects where none existed in the image itself (akin to a ‘guided hallucination’) (see the Google Research Blog article for examples^11^). Although the mechanisms employed by this simple network manipulation are not intended to be biologically plausible, the simple experiment demonstrates the power that inappropriately held prior expectations might have on resulting perception.

To what extent can inappropriately held prior expectations influence human perception? This question has relevance to descriptive psychopathology and psychiatry. Karl Jaspers, the father of descriptive psychopathology, postulated that ‘illusions due to affect’ and ‘illusions due to inattentiveness’ may arise when a person has a strong prior expectation about the state of the world and is confronted with noisy and ambiguous sensory data.^12^ This exaggerated prior expectation may be informed by the semantic context of a situation (in what have come to be termed ‘completion illusions’), the observer's current emotional state^13^ (in ‘affect illusions’), or active imaginative processes acting on inherently ambiguous sensory data (in ‘pareidolic illusions’).^1,12,14,15^ It may be argued that in these situations the observer comes to impose their prior expectations on the ambiguous sensory data.

## Contemporary accounts of hallucinations

We have argued that perceptual inference always relies on both incoming sensory data and a prior expectation about what these data are likely to represent. Additionally, we have outlined the hypothesis that illusions and hallucinations may be the result of an imbalance between these two sources of information. This simple account is consistent with contemporary theories of illusions and hallucinations, which also implicate a miscalibration between these two sources of evidence.

One of the most influential contemporary accounts of perceptual inference is that of hierarchical predictive coding.^9,16,17^ At the heart of the predictive coding account is the notion that the brain maintains a dynamic representation of the world, which is the brain's best prediction about the state that the world is likely to be in. Incoming sensory data are compared against this representation. If there is a good match between the prior prediction and the sensory data the current representation of the state of the world is reinforced. If there is a mismatch, a ‘prediction error’ signal drives an updating of the brain's current representation of the world, which is subsequently re-tested against the real-world data. The iterative process of matching the brain's predictions to sensory signals underlies perceptual inference.^10,16,17^ This process can become disrupted when the balance between prior predictions and incoming sensory data is changed. The brain's internal representation of the world will be resistant to change, and thus dominate perceptual inference, if the prior prediction is given a greater weight than the incoming sensory data, as may happen when the incoming sensory data are noisy.^9,16,17^ It has been proposed that in some pathological states the brain may mistake its own prior predictions for new incoming sensory data, resulting in perceptual and cognitive abnormalities that share some similarity to acute psychosis.^18^

Another influential account of complex visual hallucinations is the perception and attention deficit (PAD) model, which was developed after studying clinical populations who experience recurrent complex visual hallucinations.^19^ It was found that people in these populations had combined deficits in low-level sensory processing and attention. The PAD model proposes that in order to perceive an object, the perceptual object must first be selected from a pool of candidate ‘proto-objects’, in a process guided by sensory data, prior expectations and attentional processes. In people who have a combined deficit in sensory processing and attention it is conceivable that proto-objects from a misrepresentative pool become inappropriately bound to the visual scene, resulting in a hallucination.^19^

Both the predictive coding and PAD accounts of illusions and hallucinations propose that an overweighting of prior expectation relative to sensory data may underlie certain perceptual abnormalities. This overweighting may be a direct result of inappropriately held prior expectations (as can occur in states of high emotional arousal), or may be secondary to a decrease in the quality (or precision) of incoming sensory data (as may occur in states of low attention, fatigue or sensory impairment).^19,20^

## Limitations

There are several limitations and unanswered questions in this ‘expectation-based’ model of hallucinations and illusions. First, although Google's ANN provides a nice visual example of the power of overweighted prior expectation, it has key structural and functional differences when compared with the human visual system. Among these are the fact that Google's network hierarchy has many more layers than our current best estimates in the primate brain.^11,21^ Moreover, Google's network was trained to ‘see’ objects in a ‘supervised’ way, whereby it was told what the images actually represented during training. This bears little resemblance to the ‘unsupervised’ learning that occurs in the brain.

Perhaps more importantly, expectation-based accounts of illusions and hallucinations fall short of explaining some of the most frequently encountered perceptual abnormalities in clinical practice. The hallucinations recounted by patients with psychosis or organic disorders are often bizarre, and seem entirely unexpected given the environmental context.^19^ Furthermore, although the account of hallucinations given above applies to all sensory modalities, it is unclear why perceptual abnormalities often occur preferentially in one sensory modality in clinical populations (e.g. auditory verbal hallucinations in schizophrenia).^15^ These questions remain unanswered, and pose an ongoing challenge for computational accounts of perceptual abnormalities in psychiatry.

## Conclusions

Although psychiatrists ask patients about perceptual abnormalities on a daily basis, it is not often that we stop to ponder what actually distinguishes normal perceptions from perceptual distortions and deceptions. Current work in psychology, neuroscience and computer science paints a picture of normal perception as being inextricably linked to prior expectations about the state of the world. Perception depends on a delicate balance between the sensory information that we are confronted with, and the prior expectations we have about the world. If the balance is disturbed then perceptual inference becomes disrupted. Without prior expectations, perception is a mathematically ill-posed problem^4,9^ (as illustrated by Fig. 1), yet when prior expectation dominates the perceptual process, humans (and ANNs) can come to perceive objects which do not exist in the sensory data. As a result, the division between veridical perception and perceptual distortions or deceptions is more subtle than one of clear qualitative difference.
